# Experimental Study of Closed System in the Chlorine Dioxide-Iodide-Sulfuric Acid Reaction by UV-Vis Spectrophotometric Method

**DOI:** 10.1155/2011/130102

**Published:** 2011-07-26

**Authors:** Na Li, Laishun Shi, Xiaomei Wang, Fang Guo, Chunying Yan

**Affiliations:** School of Chemistry and Chemical Engineering, South Campus, Shandong University, Jinan 250061, China

## Abstract

The mole ratio *r*(*r* = [I^−^]_0_/[ClO_2_]_0_) has great influence on ClO_2_-I^−^-H_2_SO_4_ closed reaction system. By changing the initiate concentration of potassium iodide, the curve of absorbance along with the reaction time was obtained at 350 nm and 297 nm for triiodide ion, and 460 nm for iodine. The changing point of the absorbance curve's shape locates at *r* = 6.00. For the reaction of ClO_2_-I^−^ in the absence of H_2_SO_4_, the curve of absorbance along with the reaction time can be obtained at 350 nm for triiodide ion, 460 nm for iodine. The mole ratio *r* is equal to 1.00 is the changing point of the curve's shape no matter at which wavelength to determine the reaction. For the reaction of ClO_2_-I^−^-H^+^ in different pH buffer solution, the curve of absorbance along with the reaction time was recorded at 460 nm for iodine. When *r* is greater than 1.00, the transition point of the curve's shape locates at pH 2.0, which is also the point of producing chlorite or chloride for chlorine dioxide at different pH. When *r* is less than 1.00, the transition point locates at pH 7.0.

## 1. Introduction

The oxidation of iodide by chlorine dioxide was reported by Bray [1], where it was used in the analytical determination of chlorine dioxide. The kinetic study of the reaction between chlorine dioxide and potassium iodide in aqueous solution was investigated by Fukutomi and Gordon [2], where two distinctly different rates were observed in the pH range 5.5–8.5. The first reaction corresponds to the rapid formation of the intermediate [ClO_2_I^−^]. The second corresponds to the decomposition of the intermediate, which results in the formation of ClO_2_^−^ and I_2_. The reaction between iodide ion and chlorite ion exhibits a remarkable variety of kinetic phenomena [3]. Responses to single and periodic pulse perturbations have been studied experimentally and numerically by Dolnik and Epstein [4] for the reaction of chlorine dioxide and iodide ion in a stirred tank reactor. Excitability with transient oscillations was obtained for perturbations by chlorine dioxide or chlorite, while stimulation by iodide produced no excitable response.

The dynamical behavior of the chlorine dioxide-iodide reaction has been studied in a system consisting of two continuous flow stirred tank reactors (CSTRs) [5]. By introducing an additional reactant to chlorite-iodide system that can react to regenerate the iodide consumed during each cycle of oscillation, De Kepper et al. [6–8] constructed the chlorite-iodate-thiosulfate and the chlorite-iodide-malonic acid (MA) systems, which oscillate in a closed (batch) as well as an open system. The batch oscillation in the reaction of chlorine dioxide with iodine and malonic acid was studied by Lengyel et al. [9–11]. The modeling of Turing structures in the chlorite-iodide-malonic acid-starch reaction system was also further investigated [12, 13].

Illumination of the chlorine dioxide-iodine-malonic acid reaction with visible light suppresses oscillations and shifts the steady state of the reaction to lower concentrations of iodide ions [14]. In the system with starch, illumination results in a strong decrease of the steady-state concentration of the triiodide-starch complex. They suggested a simple mechanism, in which iodine atoms produced by photodissociation of molecular iodine initiate reduction of chlorine dioxide to chlorite and oxidation of iodide ions to iodine. The oxidation of iodide ion by chlorine dioxide has been studied by stopped-flow techniques at I = 1.0 mol/L (NaClO_4_) [15]. A two-term rate law was confirmed for the reaction. Strier et al. [16, 17] investigated the Turing patterns, spatial bistability, and front interactions in the [ClO_2_, I_2_, I^−^, CH_2_(COOH)_2_] reaction. The development of spiral pattern in a model representing chlorite-iodide-malonic acid reaction was investigated theoretically and numerically by Riaz and Ray [18]. A set of serially coupled flow reactors was modeled by Long et al. [19] which contain chlorite-iodide oscillators. By independently varying the reactor flow rates it is possible to produce oscillatory systems with differing periods where the ratio of the period of oscillation between reactors is always an integer value.

In a previous paper, we have studied the chlorine dioxide-iodine-malonic acid-(MA-) sulfuric acid oscillation reaction by UV-vis spectrophotometric method [20]. In the chlorine dioxide-iodine-malonic acid-sulfuric acid oscillation reaction, there are three component reactions. One of them is about the reaction of chlorine dioxide and iodide. Therefore, in this paper in order to better study the chlorine dioxide-iodide-malonic acid oscillator reaction, it is necessary to investigate the chlorine dioxide-iodide reaction at different pH range. With these considerations in mind, the purpose of this paper is to report on the influence of mole ratio *r*(*r* = [I^−^]_0_/[ClO_2_]_0_) to ClO_2_-I^−^-H_2_SO_4_ closed reaction system and pH to chlorine dioxide-iodide closed reaction system by UV-vis spectrophotometric method.

## 2. Experimental

### 2.1. Materials

Chlorine dioxide aqueous solution was prepared from sodium chlorite and diluted sulfuric acid and was purified by bubbling through 10% sodium chlorite aqueous solution to remove trace Cl_2_, then absorbed in distilled water. Stock solutions of ClO_2_ were stored in darkness at 5°C. The ClO_2_ concentration was determined by iodometric titration method. Iodine solution was prepared by dissolving iodine in distilled water and stored in darkness. The I_2_ concentration was determined by sodium thiosulfate standard solution titration analysis method. Citric acid-disodium hydrogen phosphate buffer solutions with different pH value were prepared by 0.2 mol/L Na_2_HPO_4_ solution and 0.1 mol/L citric acid solution. Sulfuric acid: 0.05 mol/L. All other chemicals were the highest purity commercially available and were used as received.

### 2.2. Methods

The reaction was started by injecting a small volume of one of the reactants into a mixture containing the other components in a spectrophotometric cell. The mixing time is about 2-3 s. Spectrophotometric measurements were performed in a TU-1800PC UV-vis spectrophotometer (Beijing Puxi Tongyong Instrument Company, Beijing, China). A complete spectrum of the reaction mixture could be obtained each second of the reaction time. In most cases, absorbances at two to three different wavelengths were used for analysis. All measurements were performed at 29°C.

## 3. Results and Discussion

### 3.1. Wavelength Measurement

**Figure 1:**
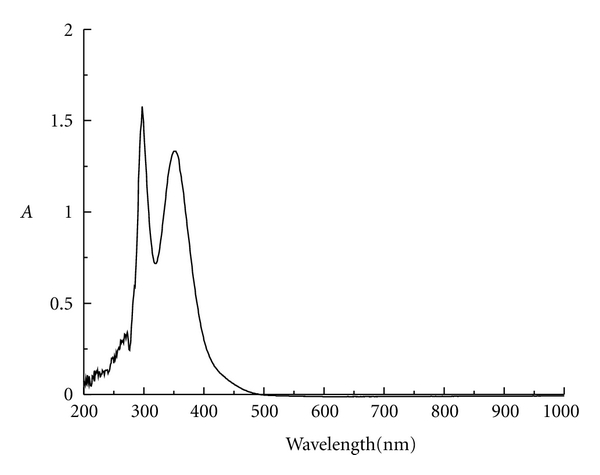
The UV-vis spectrum of triiodide ion.

The UV-vis spectra of I_3_^−^, I_2_, and I^−^ can be measured by full scanning the solution of potassium iodide reaction with iodine, the solution of iodine, and the solution of potassium iodide in the range of 200 nm–1000 nm wavelength, respectively.  Figure 1 only gives the UV-vis spectrum of triiodide ion. The maximum absorption wavelength was found to be 350 nm and 297 nm for triiodide ion. Also, we can obtain the maximum absorption wavelength to be 293 nm, 350 nm, and 460 nm for iodine and 293 nm for iodide ion, respectively.

### 3.2. The UV-Vis Spectra of ClO_2_-I^−^-H_2_SO_4_ Reaction System at Equilibrium

**Figure 2:**
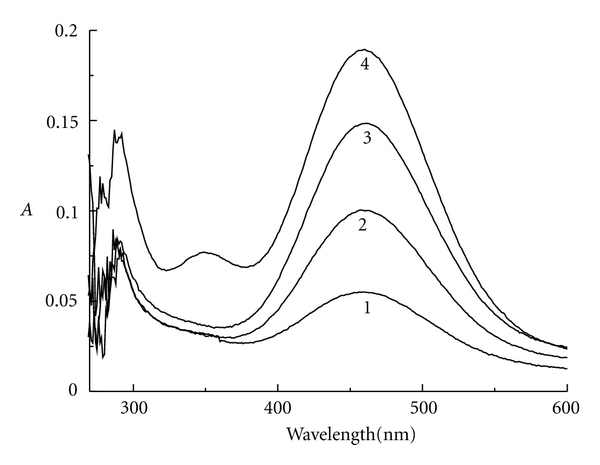
The absorbance versus wavelength at equilibrium: curve 1: *r* = 2.03; curve 2: *r* = 3.00; curve 3: *r* = 3.95; curve 4, *r* = 4.85.

For the closed reaction system of ClO_2_-I^−^-H_2_SO_4_, the reaction condition was fixed at [H_2_SO_4_]_0_ = 5 × 10^−3^ mol/L, [ClO_2_]_0_ = 1 × 10^−4^ mol/L by changing the initial concentration of potassium iodide.  Figure 2 gives the UV-vis spectra of the closed system in the range of 200 nm–600 nm when the reaction gets equilibrium. There is a strong absorption peak at 460 nm, which can be assigned to iodine. The higher the mole ratio *r*  (*r* = [I^−^]_0_/[ClO_2_]_0_) is, the stronger the peak is.

### 3.3. The Influence of Mole Ratio r(r = [I^−^]_0_/[ClO_2_]_0_) on ClO_2_-I^−^-H_2_SO_4_ Reaction

For the reaction of ClO_2_-I^−^-H_2_SO_4_, the reaction condition was fixed at [H_2_SO_4_]_0_ = 5 × 10^−3^ mol/L, [ClO_2_]_0_ = 1 × 10^−4^ mol/L by changing the initiate concentration of potassium iodide. The curve of absorbance (A) along with the reaction time can be obtained at 350 nm and 297 nm for triiodide ion. The curve of absorbance along with the reaction time can also be gotten at 460 nm for iodine.

**Figure 3:**
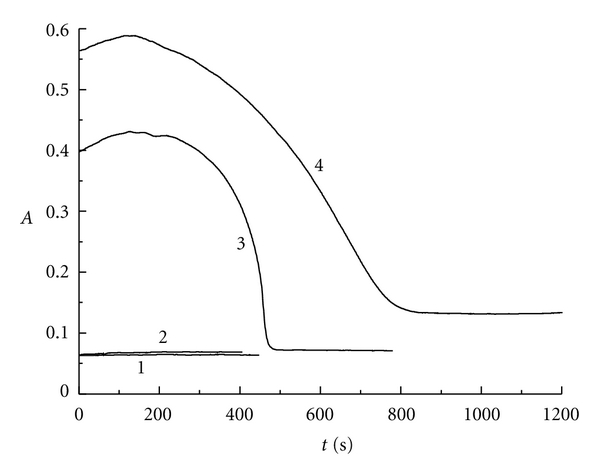
The absorbance versus reaction time at 297 nm for triiodide ion: curve 1: *r* = 2.03; curve 2: *r* = 3.00; curve 3: *r* = 3.95; curve 4: *r* = 4.85.

Figure 3 represents the absorbance changing with the reaction time at 297 nm for triiodide ion. When the mole ratio *r* is less than or equal to 3.00 (see curves 1 and 2), the absorbance does not change with the reaction time, which indicates that triiodide ion has not been produced. Under the condition that *r* is greater than 3.00 (see curves 3 and 4), the absorbance of triiodide ion changes greatly. The absorbance increases gradually at first, then decreases sharply, and finally keeps constant along with the reaction time. This phenomenon indicates that the concentration of triiodide ion has the similar changing trend with the absorbance for ClO_2_-I^−^-H_2_SO_4_ reaction system. This phenomenon can be explained as follows. At the initiate stage of the reaction, the producing ratio of iodine is greater because of the higher initiate concentration of iodide as indicated in the reaction of (R1). This also leads to the higher producing ratio of triiodide ion as indicated in the reaction of (R2). Therefore, the concentration of I_3_^−^ in the solution is higher and has maximum value at the reaction time of 130 s:

(R1) $${\text{ClO}}_{2}+5{\text{I}}^{-}+4{\text{H}}^{+}\to {\text{Cl}}^{-}+2.5{\text{I}}_{2}+2{\text{H}}_{2}\text{O}$$

(R2) $${\text{I}}_{2}+{\text{I}}^{-}\to {{\text{I}}_{3}}^{-}$$

When the reaction proceeds to certain stage, that is to say, when the reaction time is over 130 s, chlorine dioxide starts to oxidize iodide in I_3_^−^ to produce iodine as indicated in the reaction of (R3) and (R1):

(R3) $${{\text{I}}_{3}}^{-}\to {\text{I}}_{2}+{\text{I}}^{-}$$

R1 $${\text{ClO}}_{2}+{5\text{I}}^{-}+4{\text{H}}^{+}\to {\text{Cl}}^{-}+2.5{\text{I}}_{2}+2{\text{H}}_{2}\text{O}$$

At the final stage as indicated in the figure, the absorbance does not change with the reaction time, the concentration of I_3_^−^ is very low, but the concentration of iodine will be very high.

**Figure 4:**
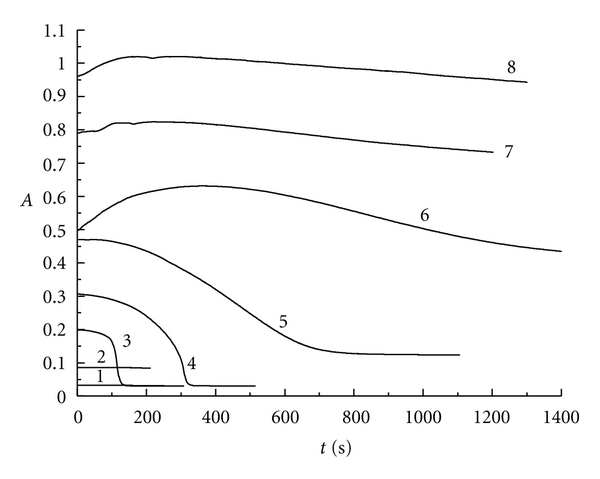
The absorbance versus reaction time at 350 nm for triiodide ion: curve 1: *r* = 1.05; curve 2: *r* = 2.03; curve 3: *r* = 3.00; curve 4: *r* = 3.95; curve 5: *r* = 4.85; curve 6: *r* = 6.00; curve 7: *r* = 7.00; curve 8: *r* = 8.00.

Similarly, Figure 4 represents the absorbance changing with the reaction time at 350 nm for triiodide ion. We can also get the same conclusion as indicated above. On the other hand, when *r* is greater than 6.00, potassium iodide in the reaction solution will be extremely excess. Therefore, the concentration of I_3_^−^ will be very high when the reaction gets equilibrium at the final stage (see curves 6, 7, and 8).

**Figure 5:**
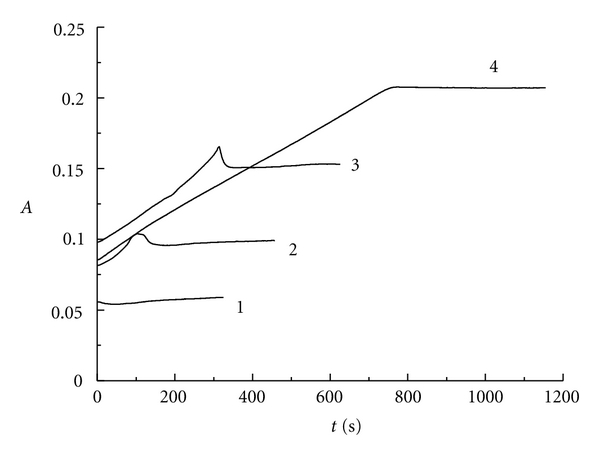
The absorbance versus reaction time at 460 nm for iodine: curve 1: *r* = 2.03; curve 2: *r* = 3.00; curve 3: *r* = 3.95; curve 4: *r* = 4.85.

Iodine has a maximum absorption peak at 460 nm. For this reason, the absorbance of iodine versus reaction time was also determined at 460 nm (see Figure 5). As shown in Figure 5 (curve 1 and curve 4), the concentration of iodine increases along with the extension of reaction time, then levels off which indicates getting reaction equilibrium. However, for curve 2 and curve 3, a small peak appears at the reaction time of 100 s and 300 s, respectively. The appearance of small peak indicates that another reaction is occurring. That is to say, this reaction leads to the decrease of iodine concentration. Also, this reaction belongs to fast reaction. It can be attributed to the reaction of (R2). This conclusion is consistent with the result of the absorbance changing trend with the reaction time at 297 nm for triiodide ion:

(R2-fast) $${\text{I}}_{2}+{\text{I}}^{-}\to {{\text{I}}_{3}}^{-}$$

### 3.4. The Influence of Mole Ratio r on ClO_2_-I^−^ Reaction System

For the reaction of ClO_2_-I^−^ in the absence of H_2_SO_4_, the reaction condition was fixed at [ClO_2_]_0_ = 1.09 × 10^−4^ mol/L by changing the initiate concentration of potassium iodide. The curve of absorbance along with the reaction time can be obtained at 350 nm for triiodide ion. The curve of absorbance along with the reaction time can also be gotten at 460 nm for iodine.

**Figure 6:**
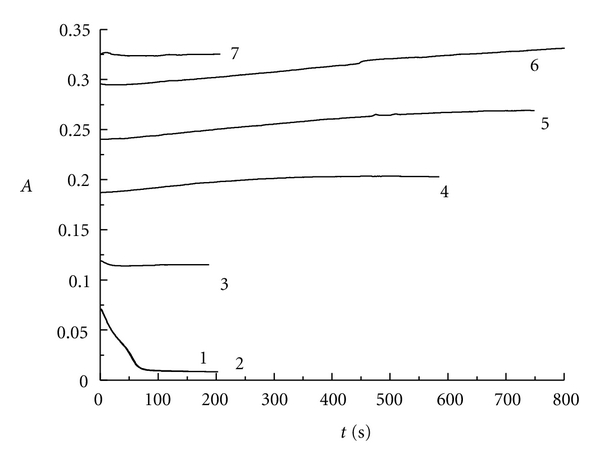
The absorbance versus reaction time for ClO_2 _-KI system at 350 nm: curve 1: *r* = 0.47; curve 2: *r* = 1.00; curve 3: *r* = 2.01; curve 4: *r* = 3.02; curve 5: *r* = 4.03; curve 6: *r* = 5.04; curve 7: *r* = 5.92.

Figure 6 represents the absorbance changing with the reaction time at 350 nm for triiodide ion. In the absence of H_2_SO_4_, that is to say, when the reaction of chlorine dioxide with iodide occurred in neutral medium, the following reaction (R4) can be occurred.

(R4) $${\text{ClO}}_{2}+{\text{I}}^{-}\to {{\text{ClO}}_{2}}^{-}+\frac{1}{2}{\text{I}}_{2}$$

One molecule of chlorine dioxide react with one molecule potassium iodide can produce one molecule chlorite and half molecule of iodine. In the neutral medium, chlorite cannot further oxidize iodide to produce iodine. The critical value of *r* for the reaction is 1.00.

When the mole ratio *r* is below or equal to 1.00 (see curves 1 and 2), the absorbance decreases along with the extension of reaction time at 350 nm and then does not change with the reaction time afterwards. Under the condition that *r* is greater than 1.00 (see curve 3 to curve 7), the absorbance increases along with the prolongation of reaction time, which indicates the increase of I_3_^−^ species concentration. It can be explained that the iodine produced by the reaction of chlorine dioxide with potassium iodide reacts with the excess iodide in the reaction system to produce I_3_^−^ as indicated by the reaction of (R2).

**Figure 7:**
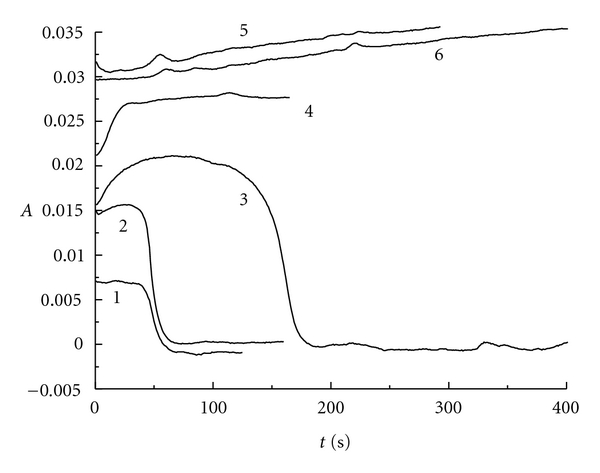
The absorbance versus reaction time for ClO_2 _-KI system at 460 nm: curve 1: *r* = 0.26; curve 2: *r* = 0.53; curve 3: *r* = 0.79; curve 4: *r* = 0.99; curve 5: *r* = 1.97; curve 6: *r* = 2.96.

At the same time, for the reaction of ClO_2_-I^−^ in the absence of H_2_SO_4_, the reaction condition was fixed at [ClO_2_]_0_ = 9.83 × 10^−5^ mol/L by changing the initiate concentration of potassium iodide.  Figure 7 represents the absorbance changing with the reaction time at 460 nm for iodine. When the mole ratio *r* is below 1.00 (see curve 1 to curve 3), the absorbance increases along with the extension of reaction time at 460 nm, then decreases sharply, and finally does not change with the reaction time. When the mole ratio *r* is equal or greater than 1.00 (see curve 4 to curve 6), the absorbance increases along with the prolongation of reaction time, which indicates the increase of iodine concentration. We can get the conclusion that the mole ratio *r* is equal to 1.00 is the changing point of the absorbance curve's shape no matter at which wavelength of 350 nm or 460 nm to determine the reaction.

### 3.5. The Influence of pH on ClO_2_-I^−^-H^+^ Reaction System at Different r

For the reaction of ClO_2_-I^−^-H^+^ in different pH buffer solutions, the reaction condition was fixed at [ClO_2_]_0_ = 9.89 × 10^−5^ mol/L, *r* = 3.26 or 0.52. The curve of absorbance along with the reaction time was recorded at 460 nm for iodine.

**Figure 8:**
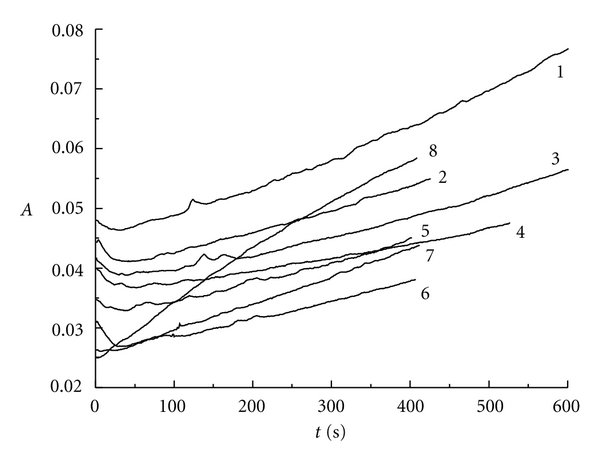
The absorbance versus reaction time for ClO_2 _-I^−^-H^+^ system at 460 nm (*r* = 3.26): curve 1: pH = 7.0; curve 2: pH = 5.7; curve 3: pH = 5.0; curve 4: pH = 4.5; curve 5: pH = 4.0; curve 6: pH = 3.6; curve 7: pH = 2.0; curve 8: pH = 1.0.

Figure 8 represents the absorbance changing with the reaction time at 460 nm for iodine (*r* = 3.26). Successive curves are shifted up by certain absorbance unit for better viewing, since in the absence of a shift the curves overlap. When *r* is greater than 1.00, the absorbance decreases slightly at first and then increases along with the reaction time in the pH range of 2.0 to 7.0 (see curve 1 to curve 6). However, in the range of pH equal to or lower than 2.0 (see curve 7 to curve 8), the absorbance increases along with the reaction time.

 As we know, when the pH is equal to 7.0,

(1) $${\text{ClO}}_{2}+{\text{e}}^{-}={{\text{ClO}}_{2}}^{-}$$

When the pH is less than or equal to 2.0,

(2) $$\begin{array}{c} {\text{ClO}}_{2}+4{\text{H}}^{+}+5{\text{e}}^{-}={\text{Cl}}^{-}+2{\text{H}}_{2}\text{O}\\ {{\text{ClO}}_{2}}^{-}+4{\text{H}}^{+}+4{\text{e}}^{-}={\text{Cl}}^{-}+2{\text{H}}_{2}\text{O} \end{array}$$

When the pH is less than 0.1,

(3) $${{\text{ClO}}_{3}}^{-}+6{\text{H}}^{+}+6{\text{e}}^{-}={\text{Cl}}^{-}+3{\text{H}}_{2}\text{O}$$

Therefore, the transition point of the absorbance curve's shape locates at pH value of 2.0. This transition point is also the right point of producing chlorite or chloride for chlorine dioxide at different pH value.

**Figure 9:**
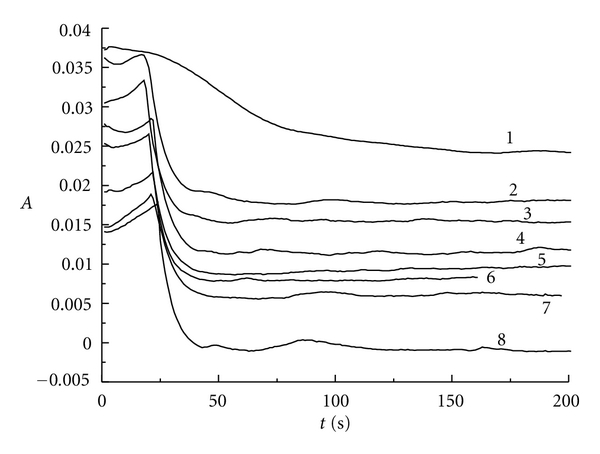
The absorbance versus reaction time for ClO_2 _-I^−^-H^+^ system at 460 nm (*r* = 0.52): curve 1: pH = 7.0; curve 2: pH = 5.7; curve 3: pH = 5.0; curve 4: pH = 4.5; curve 5: pH = 4.0; curve 6: pH = 3.6; curve 7: pH = 2.0; curve 8: pH = 1.0.

Figure 9 gives the absorbance changing with the reaction time at 460 nm for iodine (*r* = 0.52). Successive curves are also shifted up by certain absorbance unit for better viewing, since in the absence of a shift the curves overlap. When *r* is less than 1.00, the absorbance increases at first, then decreases sharply, and finally keeps constant along with the reaction time in the pH range of 1.0 to 5.7 (see curve 2 to curve 8). The sharp changing point is corresponding to the occurrence of fast reaction (R2), which leads to the decrease of iodine concentration. When the pH is equal to 7.0 (see curve 1), the absorbance decreases along with the reaction time.

Lengyel et al. studied the ClO_2_-I_2_-MA chemical oscillatory reaction system in a closed system [10]. The closed system containing an aqueous mixture of chlorine dioxide, iodine, and a species such as malonic acid (MA), which reacts with iodine to produce iodide, shows periodic changes in the absorbance of I_3_^−^ at 280 nm. This behavior can be modeled by a simple scheme consisting of three component reactions: (1) the reaction between MA and iodine, which serves as a continuous source of I^−^; (2) the reaction between ClO_2_ and I^−^, which acts as a source of ClO_2_^−^; (3) the self-inhibited reaction of chlorite and iodide that kinetically regulates the system:

(R5) $${\text{CH}}_{2}{\left(\text{COOH}\right)}_{2}+{\text{I}}_{2}\to \text{ICH}{\left(\text{COOH}\right)}_{2}+{\text{I}}^{-}+{\text{H}}^{+}$$

R4 $${\text{ClO}}_{2}+{\text{I}}^{-}\to {{\text{ClO}}_{2}}^{-}+\frac{1}{2}{\text{I}}_{2}$$

(R6) $${{\text{ClO}}_{2}}^{-}+4{\text{I}}^{-}+4{\text{H}}^{+}\to {\text{Cl}}^{-}+2{\text{I}}_{2}+2{\text{H}}_{2}\text{O}$$

Actually, reaction (R4) is one of three component processes whose kinetics depend on the pH values [20]. At pH 3.2–3.8, the closed system of ClO_2_-I_2_-MA shows periodic changes in the light absorbance of I_3_^−^ at 280 nm [20]. At even lower pH value, we cannot observe the oscillation phenomena because of the fast reaction of (R7) at lower pH value, which cannot reproduce iodine:

(R7) $$5{{\text{ClO}}_{2}}^{-}+2{\text{I}}_{2}+2{\text{H}}_{2}\text{O}=4{{\text{IO}}_{3}}^{-}+5{\text{Cl}}^{-}+4{\text{H}}^{+}$$

At even higher pH value, we also cannot see the oscillation phenomena because of the slow reaction of (R6) at higher pH value, which cannot reproduce iodine from iodide ion. The appearance of oscillation depends critically on the pH value of the solution. Therefore, the investigation of the influence of mole ratio and pH value for the chlorine dioxide-iodide closed reaction system in this paper is important for the further study of chlorine dioxide-iodide-malonic acid oscillator reaction.

## 4. Conclusion

1. The mole ratio *r*(*r* = [I^−^]_0_/[ClO_2_]_0_) has great influence on ClO_2_-I^−^-H_2_SO_4_ reaction system. By changing the initiate concentration of potassium iodide, the curve of absorbance along with the reaction time was obtained at 350 nm and 297 nm for triiodide ion. The concentration of triiodide ion increases gradually at first, then decreases sharply, and finally keeps constant along with the reaction time. When *r* is greater than 6.00, the concentration of triiodide ion will be very high when the reaction gets equilibrium at the final stage because of extremely excess potassium iodide in the reaction solution. The curve of absorbance along with the reaction time can also be gotten at 460 nm for iodine. The concentration of iodine increases along with the extension of reaction time and then levels off, which indicates getting reaction equilibrium.
2. For the reaction of ClO_2_-I^−^ in the absence of H_2_SO_4_, the curve of absorbance along with the reaction time can be obtained at 350 nm for triiodide ion by changing the initiate concentration of potassium iodide. When the mole ratio *r* is below or equal 1.00, the absorbance decreases along with the extension of reaction time and then does not change with the reaction time afterwards. Under the condition that *r* is greater than 1.00, the absorbance increases along with the prolongation of reaction time, which indicates the increase of triiodide ion concentration.

The curve of absorbance along with the reaction time can also be gotten at 460 nm for iodine. When the mole ratio *r* is below 1.00, the absorbance increases along with the extension of reaction time, then decreases sharply, and finally does not change with the reaction time. The concentration of iodine increases along with the prolongation of reaction time when the mole ratio *r* is equal to or greater than 1.00. The mole ratio *r* equal to 1.00 is the changing point of the absorbance curve's shape no matter at which wavelength of 350 nm or 460 nm to determine the reaction.

(3) For the reaction of ClO_2_-I^−^-H^+^ in different pH buffer solutions, the curve of absorbance along with the reaction time was recorded at 460 nm for iodine. When *r* is greater than 1.00, the absorbance decreases slightly at first and then increases along with the reaction time in the pH range of 2.0 to 7.0. However, in the range of pH equal to or lower than 2.0, the absorbance increases along with the reaction time. The transition point of the absorbance curve's shape locates at pH value of 2.0, which is also the right point of producing chlorite or chloride for chlorine dioxide at different pH values.

When *r* is less than 1.00, the absorbance increases at first, then decreases sharply, and finally keeps constant along with the reaction time in the pH range of 1.0 to 5.7. The absorbance decreases along with the reaction time when the pH is equal to 7.0.
